# Choosing the proper animal model for oral submucous fibrosis research: considerations and challenges

**DOI:** 10.3389/fphys.2025.1501158

**Published:** 2025-03-06

**Authors:** Linlin Zhang, Jun Chen, Fuxingzi Li, Binjie Liu, Junjie Liu

**Affiliations:** ^1^ Hunan Key Laboratory of Oral Health Research, Department of Periodontics and Oral Medicine, Xiangya Stomatological Hospital, Xiangya School of Stomatology, Hunan Clinical Research Center of Oral Major Diseases and Oral Health, Academician Workstation for Oral-maxillofacial and Regenerative Medicine, Central South University, Changsha, China; ^2^ Department of Metabolism and Endocrinology, National Clinical Research Center for Metabolic Disease, The Second Xiangya Hospital, Central South University, Changsha, Hunan, China

**Keywords:** oral submucous fibrosis, animal model, areca nut, HOCl, bleomycin

## Abstract

**Objectives:**

Animal models of oral submucous fibrosis (OSF) are essential for the studying on the pathogenesis of this disease. Current research on animal models of OSF requires further investigation. In this review, we aim to summarize the strengths and weaknesses of existing OSF animal models, as well as the recent progress in this field.

**Subject and methods:**

OSF is an oral potentially malignant disorder (OPMD) characterized by fibrotic bands, burning sensations, and limited mouth opening. Numerous experimental animal models have been developed to replicate the pathological processes in patients with OSF. Therefore, we systematically evaluated existing animal models of OSF classifying them according to the elements of building an animal model.

**Results:**

In this study, we propose that the elements of animal models for OSF include inducers, animal species, and methods of intervention. Additionally, we highlighted the advantages and limitations of these models and provided directions for future research.

**Conclusion:**

Using human-like animals as experimental subjects, combining both physical and chemical stimulation, and adjusting the dosage and type of inducer may represent the direction of future studies in this field.

## 1 Introduction

Oral submucous fibrosis (OSF) is a disease characterized by submucous fibrosis, ulceration, a burning sensation, and limited mouth opening. It was first identified as a distinct disease by Indian researchers in 1953 (Pindborg et al., 1964). It has become increasingly prevalent in Asian regions, especially in Vietnam, India, and several Chinese provinces. As a result, it has emerged as a significant global healthcare concern (McGurk and Craig, 1984; Shah et al., 2001). In mainland China, the reported prevalence of OSF ranges from 0.9% to 4.7% (Liu et al., 2015), and from 2.5% to 3.0% in India (Kumbhalwar et al., 2022), with these rates continuing to rise. OSF is a potentially malignant disorder with a high malignant transformation rate (1.5%–15%) (Reichart and Phillipsen, 1998), posing a serious threats human life. Therefore, the prevention and treatment of OSF are critical issues.

### 1.1 The etiology of OSF

It is widely accepted that the pathogenic factors of OSF include areca nut chewing (Reichart and Phillipsen, 1998; Zain et al., 1999; Lee et al., 2003; Chung et al., 2005; Oakley et al., 2005; Reichart and Nguyen, 2008), nutritional disorders (Jani et al., 2017; Sachdev et al., 2018), genetic predisposition (Chiu et al., 2002; Chen et al., 2004; Xie et al., 2012), and immunologic factors (Pillai et al., 1987; Liu et al., 2022). A large body of epidemiological, *in vivo*, and *in vitro* studies has demonstrated that betel nut is a major causative factor in the development of OSF (Lee et al., 2003; Chung et al., 2005; Hazarey et al., 2007; Maher et al., 1994; Yang et al., 2001; Yang et al., 2005; Chen et al., 2006; Yen et al., 2007). Betel nut contains numerous bioactive components, including alkaloids, flavonoids, phenolic compounds, and essential oils. Among these, the most important and widely studied component is arecoline, which is considered the primary active ingredient responsible for inducing OSF.

Key components found in betel nut include:

Arecoline: A primary alkaloid known to promote fibroblast proliferation and collagen deposition, contributing to the fibrotic process in OSF.
Arecaidine: Another alkaloid that may have similar effects to arecoline in the development of OSF.
Tannins: Polyphenolic compounds that can contribute to tissue irritation and fibrosis.
Flavonoids: Antioxidant compounds that may have both protective and harmful effects, depending on their interaction with other components.
The high concentration of arecoline in betel nut is believed to be the most significant factor in the induction of OSF, acting through various molecular pathways to stimulate fibroblasts, inhibit collagen degradation, and promote extracellular matrix accumulation, ultimately leading to fibrosis and subsequent tissue scarring. Understanding the pathogenesis and pathological characteristics of OSF is crucial. However, its exact pathogenesis remains unclear.

### 1.2 The pathogenesis of OSF

**FIGURE 1:**
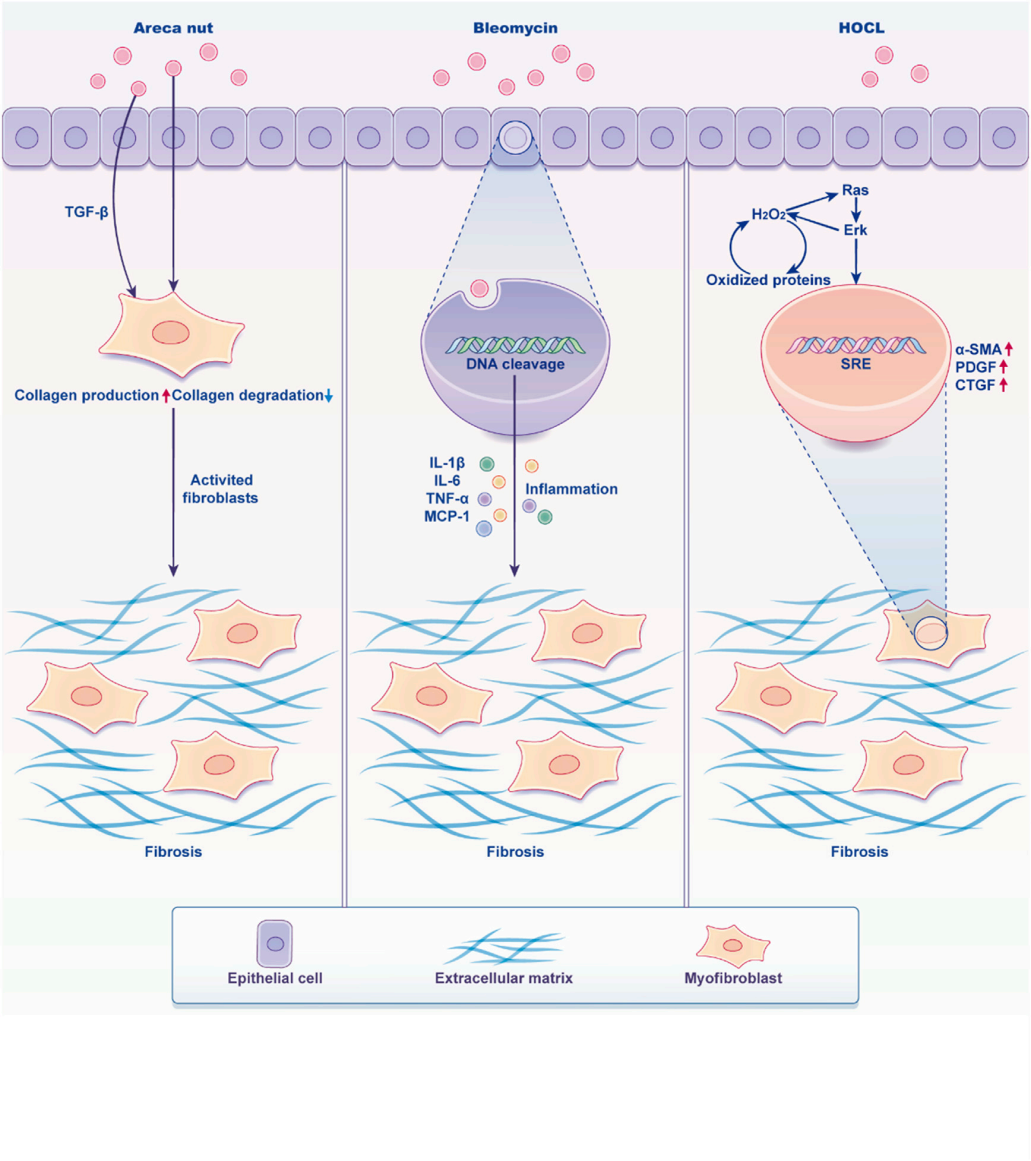
Characters of oral mucosa when stimulated by inducers. Arecoline prompts the epithelial cells of the oral mucosa to express significant levels of TGF-β1, which subsequently induces the activation of fibroblasts into myofibroblasts. This transformation results in an increased secretion of collagen fibers and reduced degradation, ultimately culminating in fibrosis. On the other hand, Bleomycin exerts a toxic effect on cells, leading to DNA fragmentation and the secretion of a large amount of cellular inflammatory factors. These factors activate fibroblasts, causing them to produce excessive collagen fibers, ultimately resulting in fibrosis. Additionally, HOCL stimulates tissues to generate a substantial amount of ROS, which enhances the phosphorylation of ERK1/2 and activates the Ras pathway and gives the diseased fibroblasts a high proliferation phenotype, leading to fibrosis. (Created by Figdraw).

Currently, the most widely accepted theories suggest that OSF pathogenesis involves abnormal expression of inflammatory cytokines and growth factors, such as TGF-β, TNF-α, IGF-1, b-FGF, and CTGF (Chiu et al., 2001; Tsai et al., 2005; Bishen et al., 2008; Khan et al., 2011; Moutasim et al., 2011; Khan et al., 2012; Chang et al., 2013; Kale et al., 2013; Yadahalli et al., 2022), abnormal activation of the NF-κB, c-Jun N-terminal kinase (JNK), and p38 MAPK pathways (Deng et al., 2009; Pitiyage et al., 2011; Liu et al., 2021), imbalance between matrix metalloproteinases (MMPs) and tissue inhibitors of matrix metalloproteinases (TIMPs) (Illeperuma et al., 2010), elevated levels of salivary copper and copper-dependent enzyme lysyl oxidase (Shieh et al., 2009), abnormal activation of the MEK, PI3K, and cyclooxygenase-2 (COX-2) signaling pathways, and the subsequent increase in heat shock protein (HSP) 47 (Utsunomiya et al., 2005; Yang et al., 2008), as well as the generation of reactive oxygen species (ROS) (Deng et al., 2009; Pitiyage et al., 2011). These changes contribute to decreased collagen degradation, increased collagen accumulation, excessive extracellular matrix (ECM) deposition and remodeling, ultimately resulting in the fibrosis characteristic of OSF. Furthermore, excessive apoptosis of endothelial cells in OSF leads to vascular endothelial damage, impairing vascular function and causing epithelial atrophy (Tseng et al., 2012). The histopathological features of OSF include epithelial atrophy, collagen fiber accumulation in the lamina propria and submucosa, reduced vascularity, and vessel occlusion (Figure 1), which collectively compromise the function and architecture of the oral mucosa.

### 1.3 The purpose of this study

The purpose of this review is to provide a comprehensive overview of the various experimental models used to study oral submucous fibrosis (OSF). Specifically, this review aims to:

Summarize Different Animal Models: Categorize and describe the various animal models based on species, inducing agents, intervention techniques, and outcome observation indicators, providing a structured comparison.
Highlight the Mechanisms of OSF: Focus on the mechanisms induced by various agents and techniques in these models, contributing to a deeper understanding of the pathogenesis of OSF.
Guide Future Research: Offer insights into future research directions, recommend optimal models for studying different aspects of OSF, and suggest improvements or new experimental designs.
Support Translation to Human Disease: Assist researchers in selecting models that best reflect the pathophysiology of OSF in humans, thereby enhancing the translational potential of experimental findings.

## 2 Method

The study was based on data obtained from a systematic search conducted on Google Scholar, PubMed, and Scopus. The search employed Medical Subject Headings (MeSH) and Boolean operators: (“oral submucous fibrosis” OR “OSF”) AND (“animal models”). No restrictions were applied regarding the year of publication.

The inclusion criteria are as follows:

1. Original experimental articles;
2. Studies involving the establishment of an animal model of oral submucous fibrosis (OSF)
3. Articles providing a detailed description of the conditions and methods used to create the OSF animal model, including the species of experimental animals, inducing agents, induction methods, induction duration, and criteria for evaluating results.

The exclusion criteria are as follows:

1. Literature where the full text is unavailable
2. Literature discussing the impact of OSF animal models on diseases affecting systems other than the oral cavity.

## 3 The existing animal models of OSF

Various animal species have been used in OSF research, including rodents (rats, mice and hamsters), rabbits, and other less commonly utilized mammals. Each species offers distinct advantages depending on the specific research objectives. In previous studies, rodents have been the most commonly used species for experimental models. Therefore, this study will focus on the application of rodent models in OSF research. The existing animal models of OSF can be classified based on inducing agents, intervention techniques and pathological tissue outcomes as follows. This classification aids in identifying the most appropriate animal model for studying specific aspects of OSF pathology and evaluating potential therapeutic interventions (Table 1).

**TABLE 1:** Existing successful animal models: classified by way of administration and animal species.

Animal species	Animal strain (gender)	No. of animals (experimental groups/control groups)	Inducers	Intervening measures	References
Mouse	BALB/c (male)	27/27	Arecoline, 500 mg/mL	Oral administration	Wen et al. (2015)
Mouse	BALB/c (male)	40/40	Arecoline, 1,000 mg/mL	Oral administration	Wen et al. (2017), Sun et al. (2021)
Mouse	BALB/c (male)	(24/24)/(24/24)	(ANE, 10 mg/mL and 20 mg/mL); (BLM, 0.5 mg/mL)	Subcutaneous administration	Chiang et al. (2016)
Mouse	Albino BALB/c (female)	20/20	ANE, 0.265 g/mL	Applying inducers to buccal mucosa	Sumeth et al. (2007)
Mouse	Swiss albino (gender unknown)	(10/10)/(10/10)	(ANE, 50 mg/mL)/(BLM)	Applying inducers to buccal mucosa and submucosal injections	Shekatkar et al. (2022)
Mouse	BALB/c (male)	12/(12/12)	(ANE, 20 mg/mL)/(ANE, 0.5 mg/mL)	Submucosal injections	Chiang et al. (2020)
Rats	Sprague Dawley (male)	40/10	ANE, 10 mg/mL	Submucosal injections	Li et al. (2006)
Rats	Sprague Dawley (female)	(10/10/10/10)/(10/10/10/10)	BLM, 1 g/mL	Submucosal injections	Zhang et al. (2016)
Rats	Sprague Dawley (male)	(6/6/6/6/6/6)/(8/8)	Arecoline, 0.5/2/8 mg/mL	Applying inducers to buccal mucosa to buccal mucosa and Mechanical stimulation	Yang et al. (2019)
Rats	Sprague Dawley (gender unknown)	10/10	ANE, 33 mg/mL	Submucosal injections	Maria et al. (2016)
Rats	Sprague Dawley (gender unknown)	(32/32)/20	ANE, 1 g/mL	Applying inducers to buccal mucosa and submucosal injections	Huang et al. (1997a), Huang et al. (1997b)
Hamsters	Unknow (male)	(28/28/25)	Powdery food containing areca nuts	Feeding	Chiang et al. (2004)
Rats	Sprague Dawley (male)	(1/1/1/1/1)	Arecoline, 10 mg/mL	Applying inducers to buccal mucosa to buccal mucosa and Mechanical stimulation	Wang et al. (2024)
Rats	Sprague Dawley (male)	(50/10)	Arecoline, unknown	Submucosal injections	Xuan et al. (2024)
Mouse	BALB/c (male)	(5/5/5/5)	Arecoline, 2 mg/mL	Submucosal injections	Zhou et al. (2024)

### 3.1 By inducing agents

#### 3.1.1 Areca nut extract

According to the International Agency for Research on Cancer (Raghavan and Baruah, 1958; Shivashankar et al., 1969; Arjungi, 1976; Betel-quid and areca-nut, 2004), the primary chemical constituents of areca nuts include carbohydrates, fats, proteins, crude fiber, polyphenols (flavonols and tannins), alkaloids, and mineral matter. While alkaloids are the most significant components, other constituents also play crucial roles in the development of oral submucous fibrosis (OSF). Research has shown that certain polyphenols can exacerbate OSF by cross-linking collagen fibers (Sharan et al., 2012). Additionally, areca nuts contain high levels of copper, which is released into the oral environment during chewing and can activate lysyl oxidase, leading to collagen cross-linking and extracellular matrix (ECM) remodeling. One study suggested that these findings indicate copper’s potential local effect on the pathogenesis of OSF(Raja et al., 2007). Therefore, in developing animal models of OSF, it is important to consider all components of areca nut extract (ANE), including alkaloids. Additionally, some researchers used (Saikia and Vaidehi, 1983; Khrime et al., 1991; Chiang et al., 2004) areca nut powder as an inducer. While the results of these experiments were not uniformly ideal, the choice of using areca nut powder inspired future studies. Feeding experimental animals with areca nut powder or applying a paste made from areca nuts to their oral mucosa mimics the process of chewing areca nuts. Therefore, these approaches could offer new directions for selecting inducers for OSF (Table 2).

**TABLE 2:** OSF model classified by inducer and animal species.

**Areca nut extract** in
Mouse (Chiang et al., 2016; Sumeth et al., 2007; Shekatkar et al., 2022; Chiang et al., 2020), rat (Li et al., 2006; Maria et al., 2016; Huang et al., 1997a; Huang et al., 1997b), hamster (Chiang et al., 2004)
**Arecoline** in
Mouse (Wen et al., 2015; Wen et al., 2017; Sun et al., 2021; Zhou et al., 2024), rat (Yang et al., 2019; Wang et al., 2024; Xuan et al., 2024)
**Bleomycin** in
rat (Zhang et al., 2016)

#### 3.1.2 Arecoline

Arecoline is the principal alkaloid found in areca nuts. Studies have demonstrated that arecoline can stimulate fibroblasts *in vitro* (Harvey et al., 1986; Jeng et al., 1996). Regarding the mechanism of arecoline-induced OSF, recent research has shown that arecoline induces the expression of several molecules, leading to an increase in plasminogen activator inhibitor-1 (PAI-1), insulin-like growth factor-1 (IGF-1), nuclear factor kappa-light-chain enhancer of activated B cells (NF-κB), and vimentin (Tsai et al., 2005; Chang et al., 2002; Yang et al., 2003; Ni et al., 2007; Chang et al., 2014), promotion of TGF-β activity (Kong et al., 2018), and depletion of cellular glutathione (GSH) (Shieh et al., 2003). These processes ultimately result in the accumulation of extracellular matrix (ECM), the primary pathological characteristic of OSF.

Furthermore, many studies have shown that arecoline promotes the progression of OSF by stimulating reactive oxygen species (ROS) generation (Pitiyage et al., 2011). Excessive reactive oxygen species (ROS) can induce apoptosis (programmed cell death) in endothelial cells, thereby compromising the integrity of blood vessels, which contributes to the pathological alterations in the oral mucosa. These findings suggest that arecoline may be a primary pathogenic factor in the development of oral submucous fibrosis (OSF).

#### 3.1.3 Bleomycin (BLM)

BLM is a glycopeptide antibiotic isolated from the fermentation broth of *Streptomyces verticillus* (Kong et al., 2018). It was initially used as an anti-cancer agent due to its cytotoxicity. Previous studies (Williamson et al., 2015; He et al., 2016) reported that BLM induces DNA oxidation, which leads to DNA strand scission, resulting in cell cycle arrest, apoptosis, and a dysfunctional repair response. Later, it was discovered that BLM could also cause severe lung and skin fibrosis (Williamson et al., 2015; Li et al., 2022). Following this discovery, researchers began using BLM to develop lung and skin fibrosis models (Luzina et al., 2013; Rangarajan et al., 2018). The animals in these studies showed pathological changes in their skin, such as the over-synthesis of ECM (Davies, 2016; Do and Eming, 2016), similar to those observed in OSF. This provided the theoretical foundation for using BLM as an inducer to create an animal model of OSF.

#### 3.1.4 Hypochlorous acid (HOCl)

Hypochlorous acid (HOCl) is a solid oxidant catalyzed and produced by the heme enzyme myeloperoxidase (MPO) (Winterbourn, 2008)*,* and it can kill pathogens under pathological conditions (Ulfig and Leichert, 2021). However, high concentrations of HOCl *in vivo* may lead to the development of several major inflammatory pathologies, including cardiovascular disease, neurodegenerative disorders, rheumatoid arthritis, chronic kidney disease, and certain cancers (Witko-Sarsat et al., 1996; Descamps-Latscha et al., 2005; Kisic et al., 2016; Aratani, 2018; Pravalika et al., 2018; Ndrepepa, 2019; Davies and Hawkins, 2020). The mechanism involves oxidative damage to proteins (Winter et al., 2008), DNA (Prütz, 1996), and lipids (Winterbourn et al., 1992). Consequently, some researchers have used HOCl to establish animal models of systemic sclerosis (SSc) (Fonteneau et al., 2017), particularly models of skin fibrosis (Ge et al., 2022; Mohammadi et al., 2022; Yamamoto et al., 2022).

Studies have shown that typical pathological fibrosis changes occur in the skin of experimental animals treated with HOCl, such as the infiltration of CD4^+^ T cells and macrophages (Gustafsson et al., 1990), and an increase in various cytokines, including transforming growth factor-β (TGF-β), interleukin-1 (IL-1), interleukin-6 (IL-6), tumor necrosis factor (TNF-α), matrix metalloproteinase-2 (MMP-2), and matrix metalloproteinase-6 (MMP-6). These changes may lead to fibroblast activation and extracellular matrix (ECM) synthesis (Ho et al., 2014). These alterations are observed in the early phase following HOCl administration. In the subsequent intermediate stage, a decrease in MMP/TIMP1 (Servettaz et al., 2009) and the loss of adipose tissue (Varga and Marangoni, 2017) could be observed, potentially resulting in (Maria et al., 2018) damage to ECM degradation and thinning of the epithelium.

Therefore, using HOCl provides valuable insights for establishing animal models of oral submucous fibrosis (OSF), as the characteristics of the intermediate and early phases are similar to the pathological changes seen in OSF. Therefore, adjusting the dosage and duration of HOCl exposure may offer a viable alternative for inducing OSF in animal models.

### 3.2 By intervention technique

Different intervention techniques include oral administration, subcutaneous injection, buccal submucosal injections, application of inducers to the buccal mucosa, or a combination of inducers and submucosal injections. Each of these methods has its own advantages and disadvantages, which are summarized below (Table 3).

**TABLE 3:** OSF model classified by intervention methods and animal species.

**Oral administration** in
mouse (Wen et al., 2015; Wen et al., 2017; Sun et al., 2021), hamster (Chiang et al., 2004)
**Subcutaneous Injection** in
rat (Chiang et al., 2016)
**Submucosal Injection** in
rat (Li et al., 2006; Zhang et al., 2016; Maria et al., 2016; Xuan et al., 2024), mouse (Chiang et al., 2020; Zhou et al., 2024)
**Topical Application of Inducing Agents to the Oral Mucosa** in
rat (Yang et al., 2019; Wang et al., 2024), mouse (Sumeth et al., 2007)
**Combination of Inducing Agents and Submucosal Injection** in
rat (Huang et al., 1997a; Huang et al., 1997b), mouse (Shekatkar et al., 2022)

#### 3.2.1 Oral administration

##### 3.2.1.1 Advantages

Non-invasive: Oral administration is simple and non-invasive, making it easier to handle animals. Systemic Exposure: Facilitates systemic exposure to inducing agents, which can replicate the chronic nature of OSF seen in humans, particularly with agents like areca nut extract or other fibrogenic substances. Mimics Human Behavior: Reflects real-world exposure, such as tobacco or areca nut chewing, which contributes to OSF in humans.

##### 3.2.1.2 Disadvantages

Variable Absorption: Absorption and bioavailability of inducing agents may vary, leading to inconsistent results. Slow Onset: Oral administration may result in a slower onset of OSF symptoms, requiring longer experimental durations.

#### 3.2.2 Subcutaneous injection

##### 3.2.2.1 Advantages

Controlled Dosage: Subcutaneous injection allows for precise control over the dosage and delivery of inducing agents, ensuring consistent exposure. Systemic Effect: Promotes systemic exposure to agents, especially those that require gradual absorption or release.

##### 3.2.2.2 Disadvantages

Invasive: Injection can cause discomfort or stress in animals, which may affect the validity of results. Local Reactions: May induce local tissue reactions, which might not accurately replicate the oral mucosa environment.

#### 3.2.3 Submucosal injection

##### 3.2.3.1 Advantages

Targeted Induction: Delivers the inducing agent directly to the site of interest, closely mimicking the localized nature of OSF in humans. Faster Onset: Induces fibrosis more rapidly, providing quicker experimental outcomes. Mimics Human Disease: Targets the oral mucosa directly, reflecting how OSF develops in humans due to areca nut chewing or other local irritants.

##### 3.2.3.2 Disadvantages

Invasive: Injections are invasive and may cause local injury, which could affect tissue integrity or influence fibrosis development. Limited to Small Areas: This method is limited to localized fibrosis in the oral mucosa, making it difficult to model systemic effects.

#### 3.2.4 Topical application of inducing agents to the oral mucosa

##### 3.2.4.1 Advantages

Non-invasive: This method is non-invasive and relatively easy to apply to animal models. Mimics Human Exposure: Direct application to the oral mucosa closely mimics how environmental factors (e.g., areca nut or tobacco) contribute to OSF in humans.

##### 3.2.4.2 Disadvantages

Limited Penetration: Topical application may not penetrate deeply enough into tissues, potentially reducing the efficacy of the model in replicating the full fibrotic process. Inconsistent Application: The area of application may not be uniform, leading to variations in the degree of fibrosis across the tissue.

#### 3.2.5 Combination of inducing agents and submucosal injection

##### 3.2.5.1 Advantages

Comprehensive Induction: This combination ensures both localized and systemic effects, leading to a more robust model of OSF. Enhanced Model Precision: Allows researchers to control both local and systemic exposure to inducing agents, making the model more accurate in mimicking human disease.

##### 3.2.5.2 Disadvantages

Invasive and Complex: This method is more invasive than single interventions and introduces additional variables that may affect outcomes. Increased Animal Stress: Multiple interventions can increase animal stress and discomfort, potentially influencing the results.

### 3.3 By outcome observation indicators

When measuring certain indicators, experimental animals may need to be euthanized to obtain tissue samples or perform invasive procedures. However, other indicators can be assessed using non-invasive detection methods, which enable researchers to monitor disease progression or physiological changes without causing harm or distress to the animals (Table 4).

**TABLE 4:** OSF model classified by outcome detection indicators and animal species.

**Histological evaluation** in
rat (Yang et al., 2019; Li et al., 2006; Zhang et al., 2016; Maria et al., 2016; Huang et al., 1997a; Huang et al., 1997b), mouse (Wen et al., 2015; Wen et al., 2017; Sun et al., 2021; Chiang et al., 2016; Sumeth et al., 2007; Shekatkar et al., 2022; Chiang et al., 2020), hamster (Chiang et al., 2004)
**Epithelial Changes** in
mouse (Wen et al., 2015; Wen et al., 2017; Sun et al., 2021; Chiang et al., 2016; Chiang et al., 2020), hamster (Chiang et al., 2004)
**Inflammatory Infiltration** in
rat (Sumeth et al., 2007), mouse (Huang et al., 1997a; Huang et al., 1997b)
**Vascular Alterations** in
mouse (Wen et al., 2015; Wen et al., 2017; Sun et al., 2021; Sumeth et al., 2007)
**Clinical Signs** in
rat (Yang et al., 2019; Li et al., 2006; Zhang et al., 2016; Maria et al., 2016), mouse (Wen et al., 2015; Wen et al., 2017; Sun et al., 2021), hamster (Chiang et al., 2004)
**Molecular Biomarkers** in
rat (Yang et al., 2019; Li et al., 2006; Zhang et al., 2016; Maria et al., 2016), mouse (Chiang et al., 2016; Shekatkar et al., 2022)
**Biomechanical Properties** in
rat (Li et al., 2006; Zhang et al., 2016; Maria et al., 2016)

#### 3.3.1 Histological evaluation

Assessment of tissue changes through histological staining techniques, such as Masson’s trichrome, Hematoxylin and Eosin (H&E) staining, or immunohistochemistry, to analyze fibrosis, collagen deposition, and inflammation. When measuring this indicator, experimental animals typically need to be euthanized to obtain tissue samples.

#### 3.3.2 Epithelial changes

Observation of epithelial hyperplasia, thinning, or atrophy as part of the disease progression. When measuring this indicator, experimental animals typically need to be euthanized to obtain tissue samples.

#### 3.3.3 Inflammatory infiltration

Presence of inflammatory cells (e.g., lymphocytes, neutrophils) in the submucosal area, particularly during the early stages of OSF. When measuring this indicator, experimental animals typically need to be euthanized to obtain tissue samples.

#### 3.3.4 Vascular alterations

Changes in blood vessels, such as thickening of the vessel walls or reduced vascularity. When measuring this indicator, experimental animals typically need to be euthanized to obtain tissue samples.

#### 3.3.5 Clinical signs

These may include restriction of mouth opening, changes in mucosal appearance (e.g., whitening, stiffness), and alterations in tissue pliability or flexibility, often assessed through clinical examination. This detection method is non-invasive and does not cause harm to the animals.

#### 3.3.6 Molecular biomarkers

Measurement of specific proteins or genes related to fibrosis, such as collagen types I and III, TGF-β, and other markers associated with fibrogenesis. The methods used for detecting these biomarkers include qPCR, Western blotting, immunohistochemistry, and other related techniques. The biological materials analyzed are typically derived from the buccal mucosa or skin tissues of the experimental animals. When measuring this indicator, experimental animals typically need to be euthanized to obtain tissue samples.

#### 3.3.7 Biomechanical properties

Quantification of the mechanical properties of the oral mucosa, such as tissue elasticity and stiffness, which are affected by the fibrotic process. This detection method is non-invasive and does not cause harm to the animals.

## 4 Discussion

After years of research, significant advances have been made in the study of animal models of oral submucous fibrosis (OSF). However, challenges persist in this field. We evaluate all current research on OSF animal models using a standardized framework (Denayer et al., 2014) to identify the most suitable cases for each intervention method and the animal models that most closely replicate human OSF (Tables 5, 6). This standardized scoring system includes the following criteria: animal species, disease induction method, face validity, and the complexity of outcome assessment indicators (Denayer et al., 2014).

**TABLE 5:** Proposed validity scoring system. Adapted from Denayer et al., 2014.

Criterion	Value	Score
Species	Human	4
	Non-human primate	3
	Non-human mammal	2
	Non-mammal	1
Disease simulation	True	4
	Complex	3
	Pharmacological	2
	No	1
Face validity	>1 core symptom	4
	1 core symptom	3
	1 symptom	2
	No	1
Complexity	*In vivo*	4
	Tissue	3
	Cellular	2
	Sub-cellular/molecular	1

**TABLE 6:** Scoring of existing OSF animal models based on the standardization framework.

References	Intervention methods	Species	Disease simulation	Face validity	Complexity	Total score
Wen et al. (2015), Wen et al. (2017), Sun et al. (2021)	Oral administration	2 (non-human mammal)	2 (pharmacological)	2 (4 symptoms)	3 (tissue)	9
Chiang et al. (2016)	Subcutaneous administration	2 (non-human mammal)	2 (pharmacological)	1 (no symptom)	3 (tissue)	10
Sumeth et al. (2007)	Applying inducers to buccal mucosa	2 (non-human mammal)	2 (pharmacological)	4 (2 core symptom)	3 (tissue)	11
Chiang et al. (2016)	Subcutaneous injection	2 (non-human mammal)	2 (pharmacological)	1 (no symptom)	3 (tissue)	8
Chiang et al. (2020)	Submucosal injections	2 (non-human mammal)	2 (pharmacological)	1 ((no symptom)	3 (tissue)	8
Li et al. (2006), Zhang et al. (2016), Maria et al. (2016)	Submucosal injections	2 (non-human mammal)	2 (pharmacological)	4 (3 core symptom)	3 (tissue)	11
Xuan et al. (2024)	Submucosal injections	2 (non-human mammal)	2 (pharmacological)	4 (2 core symptom)	4 (*in vivo*)	12
Zhou et al. (2024)	Submucosal injections	2 (non-human mammal)	2 (pharmacological)	1 ((no symptom)	3 (tissue)	8
Huang et al. (1997a), Huang et al. (1997b)	Applying inducers to buccal mucosa and submucosal injections	2 (non-human mammal)	2 (pharmacological)	1 ((no symptom)	3 (tissue)	8
Wang et al. (2024)	Applying inducers to buccal mucosa to buccal mucosa and Mechanical stimulation	2 (non-human mammal)	3 (complex)	4 (2 core symptom)	4 (*in vivo*)	13
Yang et al. (2019)	Applying inducers to buccal mucosa to buccal mucosa and Mechanical stimulation	2 (non-human mammal)	3 (complex)	3 (1 core symptom)	3 (tissue)	11
Chiang et al. (2004)	Feeding	2 (non-human mammal)	3 (complex)	2 (2 symptoms)	3 (tissue)	10

Disease simulation refers to how the disease is simulated in the study, including the realistic simulation of disease state (true): the animal model accurately replicates the pathological characteristics of the disease, including its clinical features, underlying mechanisms, and progression; the use of multiple methods to induce disease, the use of pharmacological agents, or the failure to induce the disease.

Face validity is a measure based on subjective evaluation, assessing the appropriateness or relevance of the model. In this study, face validity refers to the disease symptoms observed in the experimental animals, including core symptoms directly related to OSF, such as reduced mouth opening and the appearance of white patches on the buccal mucosa. Other general symptoms, such as weight loss and fur discoloration, are less closely related to OSF but may still be observed.

Complexity refers to the biological levels at which the outcome indicators are assessed, including the *in vivo*, tissue, cellular, subcellular, or molecular levels.

Using this scoring system, we evaluated existing rodent OSF models. The study by Wang, S.Y., et al., conducted in 2024, received the highest score. Future OSF animal model construction can reference this study and potentially improve upon it, such as by incorporating non-human primates or other model species.

Mice and rats are commonly used as experimental animals in OSF research. Mice offer advantages such as high fertility, docile temperament, genetic purity, and increased sensitivity to carcinogens. SD rats, being omnivorous like humans, also have a gentle nature and social advantages. Furthermore, these animals are small, inexpensive, and easy to care for, which contributes to their widespread use as OSF models. Hamsters, with their two cheek pouches, have a structure similar to that of humans and could be considered for future studies. Some research has also used other animals, such as New Zealand white rabbits (Song, 2009), although these studies lacked detailed experimental data, which limits their reliability.

Given the ongoing challenges in previous research, such as long experimental periods and low success rates, the proper dosage of inducers still requires further investigation and refinement. Continued efforts are necessary for advancing animal models of OSF, and we hope that our study will contribute to future advancements in this field. Although this article provides a comprehensive overview of current disease models for OSF, it does not offer a definitive conclusion on which model is optimal. However, researchers can select one or more appropriate experimental models based on their specific objectives and conditions to enhance the credibility of their research findings.

## 5 Summary and future directions

Establishing animal models is essential for studying oral submucous fibrosis (OSF); however, a universally accepted standard model does not yet exist. This may be due to a lack of consensus on the most effective inducers, animal species, and intervention methods. To address this challenge, it is crucial to explore these factors in greater depth and develop more refined strategies for future research. Given that the exact mechanisms of OSF remain unclear, further investigation and better-designed animal models are critical.

The pathogenic factors of OSF include both physical and chemical stimuli. Purely physical stimuli are insufficient to induce the development of OSF(89); however, chemical stimulation with ANE can induce typical fibrotic changes *in vivo* (Khrime et al., 1991). Endoscopic examination reveals whitening of the esophagus in some OSF patients (Misra et al., 1998), and pathological analysis shows esophageal fibrosis in approximately two-thirds of patients (Shilpa et al., 2011). These findings suggest that chemical stimuli may serve as the initiating factor, while physical stimuli play a promoting role. Therefore, we believe that only by combining both physical and chemical stimuli can an animal model that closely mimics OSF be induced.
